# Left auditory cortex is involved in pairwise comparisons of the direction of frequency modulated tones

**DOI:** 10.3389/fnins.2013.00115

**Published:** 2013-07-09

**Authors:** Nicole Angenstein, André Brechmann

**Affiliations:** Special Lab Non-Invasive Brain Imaging, Leibniz Institute for NeurobiologyMagdeburg, Germany

**Keywords:** auditory perception, dichotic listening, frequency modulation, functional magnetic resonance imaging, sequential comparison, working memory

## Abstract

Evaluating series of complex sounds like those in speech and music requires sequential comparisons to extract task-relevant relations between subsequent sounds. With the present functional magnetic resonance imaging (fMRI) study, we investigated whether sequential comparison of a specific acoustic feature within pairs of tones leads to a change in lateralized processing in the auditory cortex (AC) of humans. For this we used the active categorization of the direction (up vs. down) of slow frequency modulated (FM) tones. Several studies suggest that this task is mainly processed in the right AC. These studies, however, tested only the categorization of the FM direction of each individual tone. In the present study we ask the question whether the right lateralized processing changes when, in addition, the FM direction is compared within pairs of successive tones. For this we use an experimental approach involving contralateral noise presentation in order to explore the contributions made by the left and right AC in the completion of the auditory task. This method has already been applied to confirm the right-lateralized processing of the FM direction of individual tones. In the present study, the subjects were required to perform, in addition, a sequential comparison of the FM direction in pairs of tones. The results suggest a division of labor between the two hemispheres such that the FM direction of each individual tone is mainly processed in the right AC whereas the sequential comparison of this feature between tones in a pair is probably performed in the left AC.

## Introduction

The perception of complex auditory stimuli like speech and music requires the processing not only of single sounds but also of sequences of auditory stimuli. Further, the processing of an auditory sequence requires maintaining not only specific acoustic parameters in memory but in addition, a continuous update of the information in memory in order to process the then ongoing parts of the sequence. Such sequential comparisons, however, are fundamental for language and music processing which require extracting relative changes in a number of different acoustic parameters (e.g., duration, frequency, intensity). For example, regarding the parameter duration, identifying whether a tone is short or long always requires the comparison with at least one other stimulus. For this comparison, it is necessary to hold the information regarding the properties of one stimulus in memory until it can be compared with another stimulus. Regions in frontal, parietal, and temporal areas (e.g., auditory cortex, AC) with different lateralization have been shown to be involved in auditory pitch or verbal working memory (Zatorre et al., 1992, 1994; Gaab et al., 2003, 2006; Brechmann et al., 2007; Koelsch et al., 2009; Schulze et al., 2011; Schulze and Koelsch, 2012).

For the present study we investigated whether a process of sequential comparison of a specific acoustic feature within pairs of tones leads to a change in lateralized processing in the AC. For that, we used the active categorization of the direction of slow continuous frequency modulation (FM). We chose this categorization task because it is well-established that this task is mainly processed in the right AC in different species: gerbils (Wetzel et al., 1998a,b, 2008), rats (Rybalko et al., 2006), and human subjects (Poeppel et al., 2004; Behne et al., 2005; Brechmann and Scheich, 2005). Müller et al. (2001) showed bilateral temporal activity during the detection of one rising FM (no categorization). In all these earlier studies, the decision with regard to the direction of the FM had to be made on individual tones and did not require comparison with previous tones. For the present study we investigated which other auditory areas are recruited to complete the sequential comparison process. We hypothesized that this sequential process triggers mechanisms in the left AC. From lesion and psychoacoustic studies a left hemispheric specialization for sequential processing has long been suggested (Bradshaw and Nettleton, 1981). In addition, one study that also used FM tones found that sequential comparisons in a 2-back matching-to-sample task regarding pitch and FM direction especially involved a region in the left planum temporale (Brechmann et al., 2007).

For the present study, we used our recently introduced functional magnetic resonance imaging (fMRI) approach to investigate lateralized processing in the AC with contralateral noise (Behne et al., 2005, 2006). This method exploits the contralaterality of the auditory pathway where the representation of information from the contralateral ear dominates and input from the ipsilateral ear is suppressed (Kimura, 1967; Kaneko et al., 2003; Brancucci et al., 2004). Our method is able to reveal hemispheric specialization when information-bearing stimuli are presented ipsilateral to the specialized hemisphere (Behne et al., 2005, 2006). We proposed that the reduced signal-to-noise-ratio (SNR) during ipsilateral presentation of the task-relevant stimuli in conjunction with contralateral noise triggers compensatory mechanisms to adequately solve the task. This causes a selective increase of activity in the hemisphere that is specialized for the task at hand. Increasing activity with decreasing SNR by using masker stimuli has also been shown in other studies (Scheich et al., 1998; Wong, 2002; Scott et al., 2004; Bertoli et al., 2005). By using this method we showed that additional contralateral noise selectively increased activity in the right AC during active categorization of FM direction (Behne et al., 2005). Correspondingly, additional contralateral noise increased activity in the left AC during the processing of speech sounds (Behne et al., 2006; Stefanatos et al., 2008). The location of a significant increase in activity in an AC area signals the specific contribution of that area in the solution of a given task. The advantage of our method is that the performance of a single task on the same stimuli is sufficient to infer the lateralization of auditory cortical processing. Thus, the method requires neither additional control tasks nor other stimuli for comparison. In the present study we expected an increase in activity due to additional contralateral noise in the right AC because the evaluation of FM direction was required (Behne et al., 2005). We aimed to answer the question whether the pairwise comparison of FM direction changes this right hemispheric effect. The interpretation of the results of the study by Brechmann et al. (2007), which showed an involvement of the left AC in sequential processing of FM tones, predicts an additional engagement of the left AC in the task of the present study. We aimed to reveal this by showing an additional effect of the noise on the activity in the left AC.

## Materials and methods

### Participants

In the present study, 16 subjects with normal hearing participated [15 right-handed (Edinburgh Handedness Inventory; laterality quotient ≥ +60), one ambidextrous]. The absolute pure tone thresholds were ≤25 dB hearing level in the range of 250 Hz–4 kHz. The interaural hearing difference at each tested frequency was ≤10 dB. Participants (age 21–40 years, mean age 27 years, 11 females) gave written informed consent to the study, which was approved by the ethics committee of the University of Magdeburg. One additional participant was excluded from the final analysis because they were not able to solve the task tested with a two-sided chi-square test, with the number of hits, false alarms, misses, and correct rejections at a significance level of *p* < 0.01 (χ^2^ < 6.63).

### Stimuli and task

Complex linear FM tones with either upward or downward unidirectional modulation of frequency served as acoustic stimuli. The FM tones lasted 400 or 600 ms including linear rise/fall times of 10 ms. The complex tones consisted of five harmonics of decreasing amplitude (100% amplitude for fundamental frequency, 80% for 2nd harmonic, 60% for 3rd, 40% for 4th, 20% for 5th). The center-frequencies (*F_C_*) of the fundamentals were 380, 460, 540 … 940 Hz. The starting and end frequencies of the FM tones were calculated by *F_C_* [Hz] ± *F_C_* [Hz] × 0.5 × duration of the tone [s]. From this set of tones we created 80 different pairs with a gap of 50 ms between the two tones. Half of the pairs consisted of FM with the same FM direction and the other half with different FM directions.

**Figure 1:**
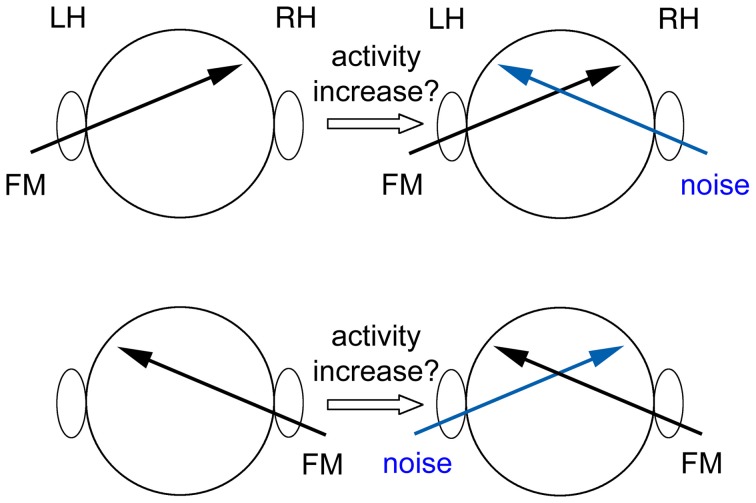
**Sketch of the experimental conditions**. The FM tones were presented either binaurally (not shown), monaurally to the right or left ear with or without contralateral white noise. Activity was compared between conditions without noise and conditions with noise. The location of the activity increase due to additional noise reveals the location of processing of the task at hand. (LH, left hemisphere; RH, right hemisphere).

During the fMRI session the tone pairs were presented in stimulation blocks of 32 s duration alternating with silence blocks of 18 s duration. Within one stimulation block, 8 pairs with same FM direction and 8 pairs with different FM directions were randomized. The blocks of tones were presented either binaurally, monaurally to the right or left ear with or without contralateral white noise (Figure 1). Five blocks for each of these five conditions were presented in pseudo-randomized order such that no two consecutive blocks belonged to the same condition. The contralateral noise was synchronized with the pairs of tones but did not contain the gap of 50 ms. The amplitude (root mean square) of the noise was 3 dB higher than the averaged amplitude of the tones.

The subjects had to compare the FM direction of the two exemplars of a tone pair and press the left button with their right index finger when the direction of the two tones was the same. For stimulus presentation and recording of behavioral responses, the Presentation software package (Neurobehavioral Systems, Albany, NY, USA) was used. Before the experiments, the overall stimulus level was adjusted for each participant to a comfortable level in the presence of scanner noise and equally loud in both ears in order to reduce potential inter-ear differences in hearing sensitivity or inter-ear differences caused by variance in the insertion of the earplugs.

### Scanning procedure

The measurements were carried out on a 3 Tesla scanner (Siemens Trio, Erlangen, Germany) equipped with an eight-channel head coil. A 3D anatomical data set of the participant's brain (192 slices of 1 mm each) was obtained before the functional measurements. Additionally, before each functional run an *Inversion-Recovery-Echo-Planar-Imaging* (IR-EPI) with the identical geometry as in the functional measurement was acquired. For fMRI 634 functional volumes were acquired in 21 min and 8 s using an echo planar imaging (EPI) sequence [echo time (TE), 30 ms; repetition time (TR), 2000 ms; flip angel, 80°; matrix size, 64 × 64; field of view, 19.2 × 19.2 cm; 30 slices of 3 mm thickness, parallel to Sylvian Fissure].

The participants' heads were fixed with a cushion with attached ear muffs containing the fMRI compatible headphones (Baumgart et al., 1998). Additionally, the participants wore earplugs. Headphones and earplugs both reduce the scanner noise by about 20–30 dB or more, depending on the frequency. All stimuli were clearly audible during the functional measurements.

### Data analysis

The functional data were analyzed using BrainVoyager™QX (Brain Innovation, Maastricht, Netherlands). A standard sequence of preprocessing steps, such as 3D-motion correction, linear trend removal, and filtering with a high-pass of three cycles per scan was performed. The head movements during the fMRI-measurement did not exceed 3 mm translation and/or 2.5° rotation. The functional data sets were projected to the corresponding IR-EPI-images, co-registered with the 3D-data set, and only for the group analysis they were transformed to Talairach-space (Talairach and Tournoux, 1988).

For the group analysis, a random-effects analysis with a general linear model (GLM), including z-transformed functional data of all 16 participants, was performed using the 2-gamma response function implemented in BrainVoyager™QX. To identify regions in the AC that were especially involved in the processing of the task, we searched for an increase in activity elicited by additional contralateral noise (Figure 1). Two conjunction analyses were performed in order to show the increase of the blood-oxygen-level-dependent (BOLD) signal due to the additional contralateral noise (*t*≥ 4, *p* < 0.002 (uncorrected for multiple comparisons), cluster threshold: 108 mm^3^):

1. left FM with and without noise > baseline AND left FM with noise > left FM without noise,
2. right FM with and without noise > baseline AND right FM with noise > right FM without noise.

The comparison with the baseline was included in order to show only regions with a significant positive deflection of the BOLD-signal during the stimulation.

For single-subject analyses, GLMs for each subject were computed without transformation to Talairach-space. For each subject, the AC was defined according to anatomical landmarks and clusters of activity elicited by stimulation as described by Brechmann et al. (2002). The defined area included planum polare, Heschl's gyrus/sulcus, planum temporale, and the anterior part of the gyrus supramarginalis. Voxels within this area activated in at least one of the five stimulation conditions (*t* ≥ 8; *q*(FDR) < 0.001) were defined as volume of interest (VOI). Within the resulting VOIs the number of activated voxels (activated volume; AV) and their mean BOLD-signal increase for each condition was computed (*t* ≥ 8, *q*(FDR) < 0.001, cluster threshold: 108 mm^3^). The AV and the signal intensity (SI) were subjected to analyses of variance (ANOVAs) with the factors side of presentation (contralateral vs. ipsilateral), noise condition (without vs. with contralateral noise), and hemisphere (left vs. right AC). *Post-hoc* two-sided paired *t*-tests were performed. The contralaterality index was computed for AV and SI by contralateral FM presentation minus ipsilateral FM presentation divided by the sum of both conditions as in our former studies (Behne et al., 2005, 2006). This index indicates activation differences within a hemisphere between the inputs from the contralateral and ipsilateral ear. The contralaterality indices were subjected to ANOVAs with the factors noise condition (without vs. with contralateral noise) and hemisphere (left vs. right AC). The contralaterality indices of the present study were compared with the results of our previous study, in which the subjects had to categorize individual tones according to their FM direction, using Mann–Whitney-tests (Behne et al., 2005).

## Results

### Behavior

The sensitivity index (*d*') as index of task performance was 2.61 ± 0.24 for left FM tones, 2.65 ± 0.26 for binaural FM tones, 2.73 ± 0.21 for right FM tones, 2.71 ± 0.3 for left FM tones with right noise, and 2.61 ± 0.23 for right FM tones with left noise. The reaction time relative to stimulus offset was 263.6 ± 18 for left FM tones, 249.4 ± 15.3 for binaural FM tones, 252.5 ± 15.4 for right FM tones, 272 ± 18 for left FM tones with right noise, and 275.2 ± 19.4 for right FM tones with left noise. ANOVAs with the factors side of presentation (left vs. right) and noise condition did not reveal significant main effects or interactions for both *d*' [*F*_(1, 15)_ < 1.5, *p* > 0.2] and reaction time [*F*_(1, 15)_ < 3.4, *p* > 0.08]. *T*-tests for the comparison of the binaural FM condition with the other conditions also did not reveal significant differences [|t|_(15)_ < 2.1, *p* > 0.05].

A comparison of the sensitivity indices of the present study with the previous study (Behne et al., 2005) also revealed no significant differences [two-sided *t*-tests: *t*_(31)_ < 0.8, *p* > 0.4].

### fMRI

**Figure 2:**
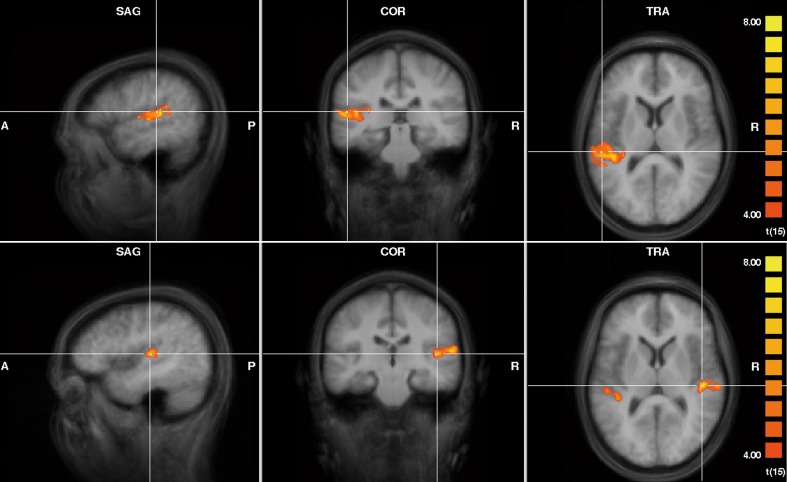
**Activity increase due to the additional presentation of contralateral noise during presentation of FM tones to the left ear (upper row; *x* = −51, *y* = −27, *z* = 12) and to the right ear (lower row; *x* = 43, *y* = −21, *z* = 10)**. Results of the group analysis of 16 subjects in Tailairach space (*t* ≥ 4, *p* < 0.002).

Group analysis showed that the additional contralateral noise during both left and right ear presentations of FM tones led to an increase in activity in the ipsilateral AC (Figure 2, *t* ≥ 4, *p* < 0.002). In addition, during presentation of FM tones to the right ear, the noise on the left ear led to an increase in activity in the left AC. The peak points of activity are shown in Table 1. According to the probability maps of Rademacher et al. (2001) and Westbury et al. (1999) the effect was located in the primary AC and in the planum temporale.

**Table 1:** **Peak points within regions with an increase of activity due to additional contralateral noise [BA-Brodmann area; *x, y, z*—Talairach coordinates, PAC—probability (%) to be localized in primary auditory cortex according to Rademacher et al. (2001), PT—probability (%) to be localized in planum temporale according to Westbury et al. (1999)]**.

**Region**	**BA**	***x***	***y***	***z***	***t***	***p***	**PAC**	**PT**
**LEFT FM WITH RIGHT NOISE > LEFT FM**								
L superior temporal gyrus	22	−48	−19	4	8.93	2 × 10^−7^	10–50	5–25
L superior temporal gyrus	41	−51	−31	10	8.2	6 × 10^−7^	0	26–65
L transverse temporal gyrus	41	−42	−31	10	8.02	8 × 10^−7^	10–40	5–45
**RIGHT FM WITH LEFT NOISE > RIGHT FM**								
R transverse temporal gyrus	41	57	−22	13	8.41	5 × 10^−7^	0	5–45
R superior temporal gyrus	41	45	−19	10	7.78	1 × 10^−6^	40–80	0
L transverse temporal gyrus	41	−36	−34	10	5.76	4 × 10^−5^	10–40	5–25
L transverse temporal gyrus	41	−48	−25	10	5.48	6 × 10^−5^	20–30	5–45

The single subject analyses revealed a main effect of side of presentation for both AV [*F*_(1, 15)_ = 73.8, *p* < 0.001] and SI [*F*_(1, 15)_ = 209.6, *p* < 0.001], a main effect of noise condition for AV [*F*_(1, 15)_ = 82.8, *p* < 0.001] and SI [*F*_(1, 15)_ = 118.7, *p* < 0.001], and a significant interaction of the factors side of presentation and noise condition for AV [*F*_(1, 15)_ = 46.8, *p* < 0.001] and SI [*F*_(1, 15)_ = 88.5, *p* < 0.001].

**Figure 3:**
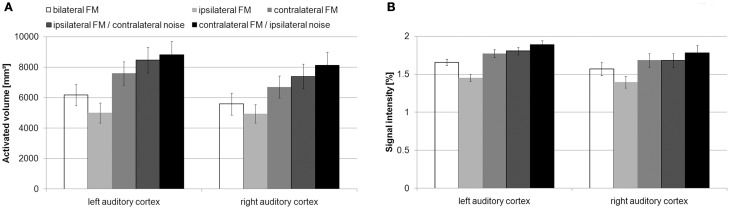
**Mean activated volume (A) and mean signal intensity (B) with standard errors within the auditory cortex during the different conditions of presentation**. In the conditions without noise, the activated volume and the signal intensity was stronger during contralateral than during ipsilateral FM presentation. Additional contralateral noise increased the activated volume and the signal intensity in left and right auditory cortex.

AV (Figure 3A) and SI (Figure 3B) in the left and right AC in the condition without noise showed the usual contralaterality with stronger activity upon contralateral than on ipsilateral stimulation [AV: left AC: |t|_(15)_ = 9.3, *p* < 1 × 10^−6^; right AC: |t|_(15)_ = 4.5, *p* < 0.001; SI: left AC: |t|_(15)_ = 16.5, *p* < 1 × 10^−10^; right AC: |t| _(15)_ = 7, *p* < 5 × 10^−6^].

The contralateral noise led to an increase in AV and SI in the left and right AC during ipsilateral FM presentation [AV: left AC: |t|_(15)_ = 9.8, *p* < 1 × 10^−7^; right AC: |t|_(15)_ = 7.7, *p* < 0.00001; SI: left AC: |t|_(15)_ = 13.5, *p* < 1 × 10^−9^; right AC: |t|_(15)_ = 8.6, *p* < 1 × 10^−6^]. With respect to our method, this indicates that both left and right auditory cortices are specifically involved in the task.

Ipsilateral noise also led to an increase in activity in the left AC [AV and SI: |t|_(15)_ < 6, *p* < 0.0001] and right AC [AV: |t|_(15)_ = 3.6, *p* < 0.005; SI: |t|_(15)_ = 3, *p* < 0.01] during the presentation of contralateral FM.

**Figure 4:**
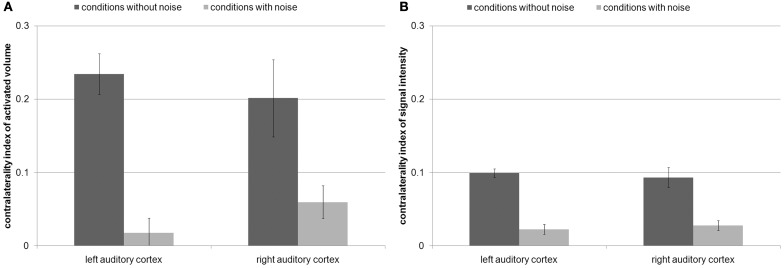
**(A)** Contralaterality index of the activated volume and **(B)** contralaterality index of signal intensity with standard errors. This index was computed by contralateral FM presentation minus ipsilateral FM presentation divided by the sum of both conditions. Positive values indicate stronger activity by contralateral FM tones than by ipsilateral FM tones. Values around zero indicate a balanced amount of activity by contralateral and ipsilateral FM tones. The contralaterality index of both activated volume and signal intensity decreased from the conditions without noise to the conditions with noise in both auditory cortices.

The ANOVAs of the contralaterality indices only revealed a main effect of noise condition for AV [*F*_(1, 15)_ = 29.6, *p* < 0.001] and SI [*F*_(1, 15)_ = 89.5, *p* < 0.001]. The contralaterality index of both AV [left AC: |t|_(15)_ = 6.3, *p* < 1 × 10^−4^; right AC: |t|_(15)_ = 3.1, *p* < 0.01] and SI [left AC: |t|_(15)_ = 10.2, *p* < 1 × 10^−7^; right AC: |t|_(15)_ = 5.4, *p* < 1 × 10^−4^] decreased from the conditions without noise to the conditions with noise in both auditory cortices (Figure 4). In other words, the addition of noise made the differences between contralateral and ipsilateral FM presentation decrease. This indicates that both left and right auditory cortices are specifically involved in the task. Nevertheless, the AV and the SI in the condition with noise were still stronger during contralateral than during ipsilateral FM presentation [AV: right AC: |t| _(15)_ = 3, *p* < 0.05; SI: left AC: |t|_(15)_ = 3.5, *p* < 0.01; right AC:|t|_(15)_ = 4.5, *p* < 0.001], except for AV in the left AC [|t|_(15)_ = 1.2, *p* > 0.2].

**Figure 5:**
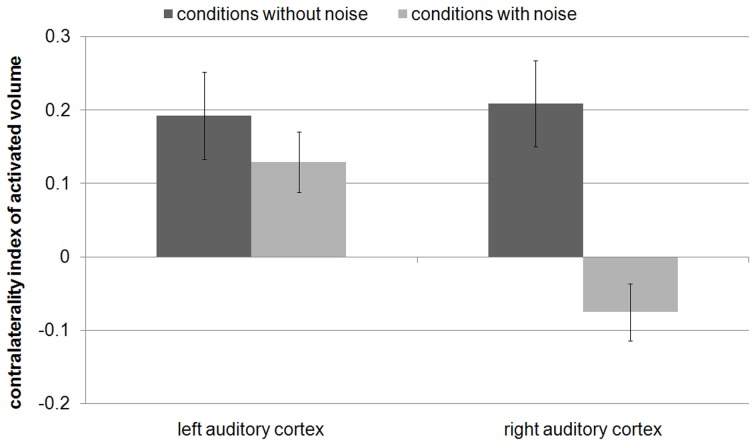
**Contralaterality index of the activated volume of our previous study (Behne et al., 2005), in which the subjects had to categorize individual tones according to their FM direction**. In contrast to the present study, the contralaterality index significantly decreased from the condition without noise to the condition with noise only in the right auditory cortex (*p* < 0.0001).

The contralaterality indices of the present study were compared (Mann–Whitney-tests) with the former study (Figure 5) in which the subjects had to categorize individual tones according to the FM direction (Behne et al., 2005). During the conditions without noise, the contralaterality indices of AV in left and right AC were not significantly different (|U|> 95, *p* > 0.1, Figures 4A, 5). During the conditions with noise, the contralaterality index in the left AC was lower in the present study than in the previous one (|U| = 70, *p* < 0.05), and in the right AC it was lower in the previous study than in the present one (|U| = 59, *p* < 0.01).

## Discussion

The results of the present study suggest that the sequential comparison of the direction of frequency modulation within tone pairs involves the AC in both hemispheres. This can be concluded from an increase of activity in left and right AC caused by additional contralateral noise during monaural ipsilateral presentation of FM tones. The result in the right AC was expected for two reasons: (1) Studies in animals and humans show that the right AC is the one mainly involved in the active categorization of FM direction without sequential comparison (Wetzel et al., 1998a,b, 2008; Poeppel et al., 2004; Brechmann and Scheich, 2005; Rybalko et al., 2006). (2) In a former study on FM categorization, the additional contralateral noise increased only the activity in the right AC (Behne et al., 2005). The difference between that study and the current one is the sequential comparison of FM direction within pairs of tones. We suggest that this additional process caused the involvement of the left AC shown in the present study.

In previous studies with contralateral noise presentation, an increase in activity caused by the noise was seen only in the hemisphere that is specialized for the task (Behne et al., 2005, 2006; Stefanatos et al., 2008): during categorization of FM direction of each individual tone, additional contralateral noise increased the activity in the right AC (Behne et al., 2005). During tasks on speech material, additional contralateral noise increased the activity in the left AC (Behne et al., 2006; Stefanatos et al., 2008). Note that all studies revealed the additional noise effect beyond the primary AC, which speaks against a pure bottom-up effect and in favor of a task-dependent effect.

There are various possible explanations for the involvement of the left AC in the task presented by this study. For the comparison within a pair, the information of the first tone has to be stored in memory until the direction of FM of the second tone is identified. In contrast, during the categorization of FM direction of individual tones, the decision can be made without referring to previous tones. Although the most relevant brain structures for working memory are in the frontal and parietal lobe (Baddeley, 2003) the AC is also involved in auditory working memory (Brechmann et al., 2007; Rong et al., 2011; Schulze and Koelsch, 2012). In an fMRI study also using FM tones by Brechmann et al. (2007), the left planum temporale was specifically involved in a 2-back working memory task concerning FM direction and pitch compared with a 0-back task. The authors suggested that the left lateralization might reflect the demand of sequential processing during the 2-back task. For that conclusion they took into account that the processing of FM direction involves mainly the right AC and that the left AC is involved in the evaluation of the duration of FM tones (Brechmann and Scheich, 2005), which also requires sequential comparisons. This also applies to the task in the present study, where the information in memory had to be continuously updated with each pair of tones presented. Such a left hemispheric specialization for sequential processing has long been suggested based on lesion and psychoacoustic studies and may explain some aspects of left lateralized speech processing (Bradshaw and Nettleton, 1981). Results showing a specialization of the left AC in active stream segregation of our laboratory were also interpreted in this framework (Deike et al., 2004, 2010). In another study, intracerebral evoked potentials to syllables showed sequential processing only in the left Heschl's gyrus and planum temporale and not in the right hemisphere (Liegeois-Chauvel et al., 1999). In addition, results of a pitch memory task (Gaab et al., 2003, 2006) suggested that the supramarginal gyrus (SMG) is important for the short-term storage of auditory stimuli fit into this scheme. The region that they found activated in their studies partly overlaps with the regions in the AC that we found to be especially involved during the task of our current study. They revealed a greater increase in activity in the left SMG during learning in a group of strong learners compared with a group of weak learners (Gaab et al., 2006). In addition, they found a correlation of activity with the task performance in the SMG in both hemispheres but this correlation was stronger on the left side (Gaab et al., 2003). Furthermore, a magnetoencephalography (MEG) study also revealed a task-specific asymmetry in activity during an auditory delayed match-to-sample task (Rong et al., 2011). They found a left-lateralized suppression effect to the second stimulus in a sound pair of tonal contours during the performance of this task, compared with passive listening and counting.

The involvement of the left AC in the present study could also be explained by a limited amount of processing capacity within the right AC. According to a theory by Banich (1998) a division of labor between hemispheres could be useful when the processing demand is high and the capacity of one hemisphere is exceeded. This hypothesis derived from visual studies also holds for auditory processing (Scalf et al., 2009). It is possible that in the present study the direct comparison of FM direction between tones increases the task demand in such a way that the left AC becomes strongly involved in a task which is in principle processed in the right AC. However, the absence of significant differences in the sensitivity indices between the present study and the previous study by Behne et al. (2005), in which sequential comparison was not necessary, calls this explanation into question. It is also questionable whether the present task is too simple to reach the limits of the processing capacity of one hemisphere, compared with, for example, complex auditory stimuli that are perceived daily, such as speech.

According to the interpretation of the results of a MEG study by Nahum et al. (2009), the short interval between the tones within the pairs could have been the reason for the additional left hemispheric involvement in the present study. Nahum et al. (2009) found stronger responses in the left than in the right hemisphere when subjects had to decide whether the frequency of 50-ms pure tones within pairs with 100-ms interstimulus intervals was the same or different. They suggested that the short interval between the tones within the pairs requires the processing of rapidly presented stimuli and therefore leads to a stronger left hemispheric involvement in the processing of the task. It is questionable whether the presentation in our study with an overall duration of the stimulus pairs of at least 850 ms is interpretable as a rapid presentation relative to the pairs of 200 ms in the study by Nahum et al. (2009). Instead, the hypothesis that sequential comparison of tones leads to a left AC involvement may equally well explain the finding of Nahum et al. (2009).

Whatever explanation is correct, the involvement of the left AC in the present task seems to be so strong that the additional noise presented to the contralateral (right ear—noise; left ear—FM tones) as well as the ipsilateral ear (left ear—noise; right ear—FM tones) led to an increase in activity in the left AC. Such an increase in activity caused by the additional noise in the ipsilateral AC relative to where the noise was presented was not found in previous studies employing this method (Behne et al., 2005, 2006; Stefanatos et al., 2008). Thus, it supports the view that the effect of the additional noise on the activity in the AC using this method is not simply caused by bottom-up effects. Second, we suggest that this result emphasizes the strong involvement of the left AC when FM direction needs to be compared between two tones although the underlying task of determining FM direction is mainly processed in the right AC.

## Conclusion

The results of this study suggest that the pairwise comparison of the direction of FM tones involves the left AC in addition to the known involvement of the right AC in processing FM tone direction. This means that the need to compare a characteristic feature between tones drives the involvement of the left AC although the main processing of the feature mainly takes place in the right AC. Further experiments are necessary to clarify whether the involvement of the left AC is actually caused by the continuous updating of information in memory or by an overall increase in task complexity. Future research should also determine whether the involvement of the left AC in pairwise comparisons of FM direction can be generalized for other sounds and other properties as well.

### Conflict of interest statement

The authors declare that the research was conducted in the absence of any commercial or financial relationships that could be construed as a potential conflict of interest.
